# Effects of creatine supplementation on exercise performance, physiological outcomes, and body composition in females engaged in exercise or training: a systematic review and multilevel meta-analysis

**DOI:** 10.3389/fnut.2026.1921827

**Published:** 2026-09-04

**Authors:** Yi Lin, Wenfeng Zhu, Banghui Hong

**Affiliations:** 1 School of Physical Education, Guizhou Normal University, Guiyang, China; 2 College of Physical Education and Sport Science, Fujian Normal University, Fuzhou, China

**Keywords:** body composition, creatine, exercise performance, female athletes, females engaged in exercise or training, multilevel meta-analysis, physiological outcomes, systematic review

## Abstract

**Background:**

Female-specific creatine trials span different sports, training states and outcome types. The preceding systematic review included 27 studies and provided a narrative synthesis of performance outcomes; the average magnitude of effects across correlated outcomes remained unknown.

**Objective:**

To estimate overall and domain-specific effects of creatine supplementation on exercise and sport performance, physiological outcomes and body composition in females engaged in exercise, sport or structured training.

**Methods:**

PubMed, Scopus, Web of Science, SPORTDiscus and CINAHL were searched for experimental studies published from 1 January 1996 to 5 March 2026. Two reviewers independently screened records, extracted data and appraised risk of bias. Female-specific creatine-vs.-control contrasts were synthesized with multilevel random-effects models and CR2 small-sample robust inference. Sensitivity analyses addressed graph-digitized and reconstructed percentage-change data and plausible within-study sampling correlations. GRADE was applied to the overall estimate and each quantitative domain. The review was retrospectively registered on OSF (https://doi.org/10.17605/OSF.IO/U8V7A).

**Results:**

The search identified 13,244 records; 160 reports were assessed in full text, and 34 studies with 692 female or female-eligible participants entered the systematic review. Twenty-one studies contributed 170 model-compatible effects from 424 analyzable participants. The overall model yielded a small positive estimate (Hedges g = 0.23, CR2 robust 95% CI 0.09 to 0.37). The performance model yielded g = 0.30 (95% CI 0.06 to 0.54), and the body-composition model yielded g = 0.24 (95% CI 0.06 to 0.43). The body-composition model combined outcomes without a uniform favorable direction. The physiological estimate was uncertain (g = 0.10, 95% CI −0.17 to 0.38). Risk-of-bias concerns and variation in protocols and outcomes reduced certainty, which was low for performance and body composition and very low for the overall and physiological evidence.

**Conclusion:**

Available female-specific evidence suggests possible small effects on selected strength, power, high-intensity performance and lean-mass-related measures. Substantial heterogeneity, methodological limitations and low or very-low certainty prevent firm sport-specific or outcome-specific conclusions.

**Systematic review registration:**

https://doi.org/10.17605/OSF.IO/U8V7A, identifier: OSF.IO/U8V7A.

## 1 Introduction

Creatine supplementation is among the most extensively studied nutritional strategies in exercise science. Evidence from adult populations links supplementation to high-intensity exercise capacity, strength and lean-mass-related outcomes (1–6). The phosphocreatine system supports rapid ATP resynthesis during short-duration, high-intensity work (7–9). Studies of creatine transport, intramuscular availability and muscle-creatine measurement support biological plausibility and indicate that response may vary with baseline status, diet and training context (10–14). Most practical evidence still comes from mixed-sex or male-dominant trials (15, 16), while broad reviews combine different populations, training contexts, formulations and endpoints (17–19).

Female athletes and women in structured training face nutritional and body-composition constraints that shape supplement use and interpretation (20, 21). Women-focused reviews identify dietary creatine exposure, baseline intramuscular availability and reproductive-hormone context as plausible sources of response variation (22–25). Estrogen-related changes in substrate use, fluid regulation and neuromuscular function may alter the physiological setting in which creatine is used; menstrual phase, oral-contraceptive use and hormonal transitions are rarely reported in trials. Direct evidence that these factors modify creatine response remains limited. Supplement-use patterns and safety observations in female athletes add practical context (26–28).

The review question covers four outcome domains with distinct meanings. Exercise and sport performance comprises direct task outputs such as strength, power, speed, time, work and sport-specific tests. Physiological outcomes comprise biomarkers and cardiorespiratory, metabolic or neuromuscular responses that are not direct performance endpoints. Body composition includes body mass, fat mass, lean or fat-free mass and body-water measures. Cognition and reaction time were retained as secondary outcomes when reported. These domains extend the performance categories used in previous female-focused work and reflect outcomes routinely collected in creatine trials, including strength and power (29, 30), repeated-sprint and sport-specific performance (31, 32), menstrual-cycle-related responses (33) and body composition (34).

The female exercise and training trial literature now spans resistance training, competitive sport and laboratory exercise. Resistance-training studies have reported female-specific strength or body-composition outcomes (35–37). Sport-specific studies have examined soccer, futsal, swimming, volleyball, dance and wrestling (31, 34, 38–42). Laboratory and training studies have evaluated anaerobic capacity, fatigue, muscle function and exercise adaptation (43–48). Some trials reported benefits, while others reported small, null or context-dependent effects (49, 50).

Tam and colleagues searched five databases through July 2024 and included 27 studies published from 1996 to 2023, with participants ranging from recreationally active to elite (51). Performance outcomes were classified as strength or power, anaerobic, or aerobic. No meta-analysis was performed because participant characteristics, protocols and performance tests varied. That review did not estimate pooled effects, model multiple outcomes reported by the same participants, or synthesize body-composition, physiological and cognitive domains as separate quantitative constructs. Its conclusion that most individual studies showed no improvement therefore left the average magnitude and precision of female-specific effects unresolved. The present search extends the evidence base through 5 March 2026 and broadens the synthesis to four outcome domains.

Multiple endpoints create a scientific problem because each study can address several performance, physiological and body-composition questions in the same participants. Selecting one endpoint per study discards eligible evidence and makes the result depend on endpoint choice. Treating every endpoint as independent overstates precision. Multilevel meta-analysis retains these outcomes within their study structure (52), and CR2 small-sample inference accounts for clustering when the number of studies is limited (53, 54). This approach can identify small average and domain-specific effects that a count of statistically significant study findings cannot quantify.

We systematically reviewed creatine supplementation studies in females engaged in exercise, sport, structured training or exercise-testing contexts published from 1 January 1996 to 5 March 2026. The full qualitative evidence base was separated from the subset suitable for quantitative synthesis. We estimated a global directional summary across eligible outcomes and construct-specific estimates for exercise and sport performance, physiological outcomes and body composition. The analyses addressed the magnitude of female-specific effects, their distribution across domains and the extent to which study design, reporting and outcome multiplicity constrain interpretation.

## 2 Materials and methods

### 2.1 Design and reporting

This study was designed as a systematic review and multilevel meta-analysis of creatine supplementation in females engaged in exercise, sport, structured training or exercise testing. The review addressed whether supplemental creatine changes exercise performance, physiological outcomes or body composition compared with placebo or a non-creatine control. Reporting followed PRISMA 2020 principles (55). The completed PRISMA 2020 for Abstracts and PRISMA 2020 checklists are provided as Supplementary Tables S1, S2, respectively. The review was retrospectively registered on the Open Science Framework after completion of the primary synthesis (registration DOI: https://doi.org/10.17605/OSF.IO/U8V7A).

### 2.2 Search strategy and eligibility criteria

PubMed, Scopus, Web of Science, SPORTDiscus and CINAHL were searched on 5 March 2026 for records published from 1 January 1996 to 5 March 2026. The search reproduced the broad concept used by Tam, Mitchell and Forsyth: (creatine) AND (woman OR women OR female) AND (exercise OR performance OR endurance OR aerobic OR strength OR power OR anaerobic OR physique OR body composition) (51). The lower date bound was selected because the preceding inception search identified 1996 as the first year with an eligible experimental report. Database-specific field tags, exact strings, limits and record counts are provided in Supplementary Table S3.

Eligible studies were experimental human studies of supplemental creatine. For screening, an eligible exercise or training population met at least one of the following author-reported descriptions: athlete or competitive sport participant, recreational sport participant, physically or habitually active participant, resistance-trained participant, or participant enrolled in a structured exercise-training program. Sedentary or previously untrained women were eligible only when the intervention assigned a repeated, structured training program. Exercise-testing studies were eligible when a standardized physical task quantified performance or an exercise-related physiological response. A minimum activity-frequency or metabolic-equivalent threshold was not imposed because most older reports did not provide comparable activity-volume data. Eligible comparisons were placebo, no-creatine control or another non-creatine condition relevant to the review question.

Studies were excluded when the intervention was unrelated to exercise, sport, structured training adaptation or exercise-performance testing, when the intervention was not creatine supplementation, when the comparator did not address the review question, or when the article did not report original experimental data. Clinical or disease-specific populations were excluded unless the condition was not the focus of the intervention question and the study otherwise addressed an eligible exercise or training context. Full-text reports that could not be retrieved or independently verified were excluded from the final evidence set.

### 2.3 Study selection and data extraction

Records were imported into EndNote 2025 for reference management, duplicate removal, title and abstract screening, and full-text decision tracking. Duplicate candidates were identified with DOI, PMID, title plus publication year, and title-only matching for long titles, followed by manual verification. Two reviewers independently screened titles and abstracts and then assessed full texts. Disagreements were resolved by discussion until consensus was reached.

Two reviewers independently extracted study and participant characteristics, creatine protocol, comparator, assessment time point, sample size, means and variance data. Outcome domains were assigned before quantitative modeling. Direct task outputs were classified as exercise and sport performance; metabolic, cardiorespiratory or neuromuscular responses without a direct task endpoint were classified as physiological; mass, fat, lean-tissue and body-water measures were classified as body composition; cognition and reaction time were secondary outcomes. Each eligible outcome or time point was retained as a separate effect-size row.

For mixed-sex studies, female-specific data were accepted only when sex-stratified sample sizes, means and variances were reported in the article or a publicly available supplementary file. When sex-stratified values were available for selected outcomes, only those outcomes entered the quantitative dataset; remaining eligible findings were retained in the narrative synthesis. Combined-sex estimates were not decomposed. Study authors were not contacted because a uniform published-data rule was applied across three decades of reports. This decision may have reduced the number of extractable female-specific contrasts.

Hedges g was used because outcomes were reported on different scales (56). Direction was assigned from an outcome-level codebook before model fitting. Higher strength, power, work, speed, endurance, lean mass and fat-free mass values were positive; time, fatigue, fat mass and body-fat outcomes were sign-reversed when lower values indicated improvement. Measures without an intrinsic favorable direction, including body mass, total body water and resting physiological measures, retained their reported increase direction and were interpreted according to the underlying construct. Female-specific change scores were preferred. Post-intervention means and standard deviations were used when change-score variance was unavailable and baseline values were sufficiently comparable. Standard errors were converted to standard deviations with the arm sample size.

For rows reported as baseline means plus percentage change, post-intervention means were reconstructed as baseline mean × (1 + percentage change/100). The baseline standard deviation was carried forward when a post-intervention standard deviation was unavailable. These rows were flagged because the reconstruction does not estimate pre-post covariance and were removed in a dedicated sensitivity analysis. The swimming outcome reported only graphically in Leenders et al. (57) was digitized from the published figure and checked against the source image. Because these values were graph-derived, the outcome was included only in a sensitivity analysis.

Four crossover studies were retained in the systematic review and excluded from the primary independent-arm model. Their reports did not provide the within-participant correlations or paired-difference variances required for a compatible standardized effect. Correlation imputation was not used because washout periods, outcome types and repeated-measures structures differed across these studies, leaving no defensible common value. Their findings and source data were retained for narrative synthesis. This conservative rule can reduce quantitative coverage when crossover reports lack paired data.

### 2.4 Risk of bias and certainty of evidence

Two reviewers independently assessed risk of bias for all studies included in the systematic review. RoB 2 was used for randomized or allocated controlled trials (58), including placebo-controlled and no-placebo controlled trials; the explicitly non-randomized experimental study was appraised with additional attention to ROBINS-I domains of confounding and participant selection. The five RoB 2 domains were the randomization process, deviations from intended interventions, missing outcome data, outcome measurement and selection of the reported result. Crossover or repeated-measures studies were assessed with attention to design-specific concerns, including period structure, carryover risk, allocation sequence and outcome completeness. Disagreements were resolved by discussion.

Certainty of evidence was rated with GRADE for the overall quantitative estimate and separately for exercise and sport performance, body composition and physiological outcomes (59). Each rating treated the contributing studies as the evidence units; multiple effect sizes from one study did not increase the study count or independently improve certainty. Risk of bias was judged across contributing studies, inconsistency incorporated residual heterogeneity and dispersion, imprecision used the CR2 robust confidence interval, and indirectness reflected the population, intervention and outcome scope of each model. Publication-bias evidence was exploratory.

### 2.5 Statistical analysis

Analyses used R 4.6.0, metafor 5.0-1 and clubSandwich 0.7.0 (52, 60, 61). The primary restricted maximum-likelihood model included a random intercept for study and a second random intercept for effect size nested within study (random = ~ 1 | study/effect). Outcome domain was examined through stratified models and was not specified as a third random level. Random slopes were not fitted because the primary model was intercept-only and contained 21 studies. Published reports did not provide sampling correlations among outcomes from the same participants, so the primary sampling-variance matrix was diagonal. CR2 robust standard errors were clustered by study with Satterthwaite degrees of freedom. CR2 protects coefficient inference against within-study clustering, while the missing sampling covariances remain unknown. Sensitivity models imposed compound-symmetric within-study sampling correlations of 0.30, 0.50 and 0.80.

The primary model included all extractable female-specific creatine-vs.-control contrasts suitable for quantitative synthesis. Its purpose was a global directional summary of eligible outcomes after outcome-specific sign coding; construct-specific interpretation rested on the domain models. Multi-arm studies were included only when the creatine- vs.-control comparison could be represented without reusing a shared comparator as an additional independent contrast. Domain-specific models were fitted for exercise and sport performance, body composition and physiological outcomes. Cognitive and reaction-time effects remained secondary components of the overall model because only two studies contributed them. Formal meta-regression was not fitted because the available study count could not support simultaneous evaluation of several correlated protocol and population characteristics.

Sensitivity analyses included the duplicate-digitized Leenders outcome, removed reconstructed percentage-change rows, removed effects with absolute Hedges g greater than 3, omitted one study at a time, and imposed within-study sampling correlations of 0.30, 0.50 and 0.80. A *post hoc* descriptive analysis stratified studies by supplementation duration (≤ 7 days or >7 days); no test of between-stratum difference was performed. Study-level inverse-variance aggregates were used for the forest plot, a conventional random-effects sensitivity estimate and the funnel plot. Funnel asymmetry regression was applied to the 21 study-level aggregates and interpreted descriptively because each aggregate combined dependent outcomes and the test had limited power.

### 2.6 Data integrity and reporting safeguards

Several safeguards kept the systematic-review and meta-analysis datasets aligned. The systematic-review set, quantitative-synthesis set and sensitivity-analysis set were tracked as separate study groups. Studies remained in the systematic review when they met the eligibility criteria but could not be harmonized for the primary quantitative model. This separation prevented narrative-only, graph-digitized and design-complex studies from being counted as primary multilevel meta-analysis studies.

For quantitative synthesis, each effect-size row retained the study identifier, outcome domain and label, time point or condition, sample size, source data type and synthesis-use flag. Direction codes were checked before model fitting. Reconstructed percentage-change rows, graph-digitized rows and extreme effect-size rows carried explicit flags for sensitivity analysis. Study-level values were calculated for visualization and study-aggregate diagnostics and were not used in the primary multilevel inferential model.

Counts reported in the PRISMA flow, Results text, tables and figure legends were checked against the screening and extraction files. The systematic-review count refers to the 34 included studies, whereas the primary quantitative count refers to the 21 studies and 170 effects that met the model-compatibility criteria. Participant counts were reported separately for the systematic-review evidence base and for analyzable creatine- vs.-control contrasts to avoid conflating eligible female participants with the subset contributing primary model effects.

## 3 Results

### 3.1 Study selection

**Figure 1:**
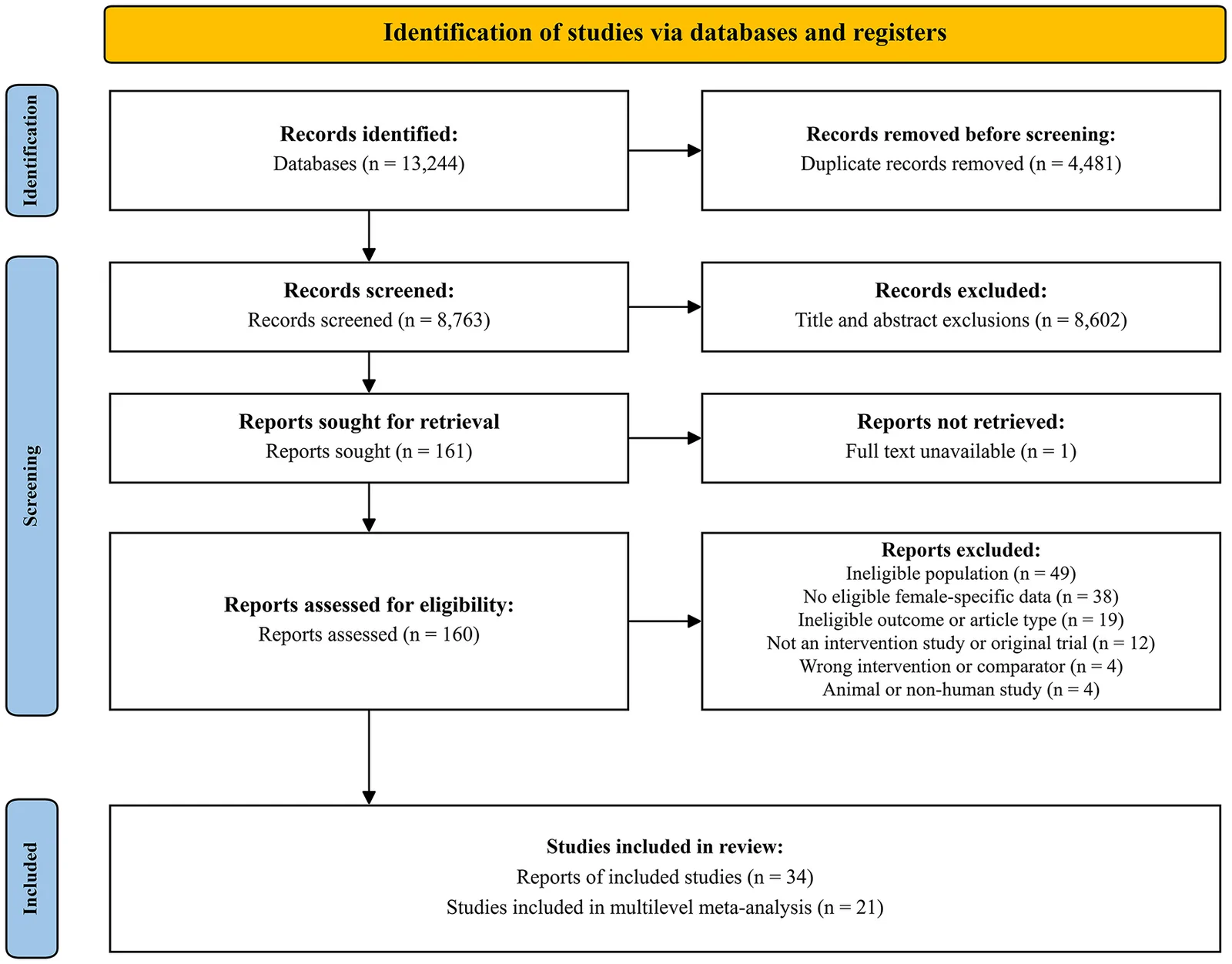
PRISMA 2020 flow diagram. Flow of records through database searching, duplicate removal, title and abstract screening, full-text assessment, and final inclusion in the systematic review and multilevel meta-analysis.

The database search identified 13,244 records from PubMed, Scopus, Web of Science, SPORTDiscus and CINAHL. After removal of 4,481 duplicate records, 8,763 records underwent title and abstract screening. A total of 161 reports were sought for full-text retrieval. One report could not be retrieved, and 160 reports were assessed for eligibility (Figure 1).

After full-text assessment, 126 reports were excluded. Thirty-four studies were included in the systematic review. Of these, 21 studies contributed 170 effect sizes to the primary multilevel meta-analysis (Figure 1). The separation between systematic-review inclusion and quantitative synthesis was retained throughout the Results because several eligible studies addressed the review question but did not provide female-specific or model-compatible contrasts for the primary analysis.

### 3.2 Characteristics of the systematic-review evidence base

The 34 included studies were published between 1996 and 2026 (Table 1). Across included reports, the systematic-review evidence base comprised 692 female or female-eligible participants. The evidence base included female athletes, recreationally active females, resistance-trained females and women enrolled in structured training or exercise-testing contexts across swimming, soccer, volleyball, futsal, dance, wrestling, resistance training and laboratory exercise settings. Most studies enrolled female-only or female-focused cohorts. Several mixed-sex studies were retained in the systematic review when female participants were eligible but female-specific quantitative data were incompletely reported.

**Table 1:** Characteristics of studies included in the systematic review.

Study	Country	Design	Population (sport/caliber)	Female/ eligible *n*	Age	Creatine form
Thompson et al. (70)	UK	RCT	Swimming/university	10	NR	Not specified
Grindstaff et al. (64)	USA	RCT	Swimming/regional to national	11	15.3 ± 0.6	CrM
Vandenberghe et al. (35)	Belgium	Double-blind controlled trial	Sedentary young females/resistance training	19	19–22	CrM
Kirksey et al. (65)	USA	Double-blind RCT	Track and field/collegiate	20 eligible women within mixed cohort	19.9 ± 0.4 by treatment group	CrM
Ledford et al. (71)	USA	Double-blind counterbalanced crossover	Recreational	10 enrolled; 9 analyzed	27.0 ± 6.0	CrM
Leenders et al. (57)	USA	RCT	Swimming/university	14	PL 19.4; CrM 19.1 (SE 0.8, 0.5)	Creatine; form not reported
Brenner et al. (36)	USA	RCT	Lacrosse/NCAA division I	16	CrM 18.1; PL 19.5 (SE 1.7, 1.9)	CrM
Haff et al. (66)	USA	Double-blind RCT	Track and field/collegiate	20 eligible women within mixed cohort	NR; treatment groups 19.9 ± 0.4	CrM
Hamilton et al. (43)	USA	RCT	Throwing/overhand sports, recreational to competitive	24	PL 23.9; CrM 22.5 (SEM 0.9, 0.8)	CrM
Larson-Meyer et al. (38)	USA	RCT	Soccer/university	14	PL: 19.0 ± 1.5; CrM: 19.3 ± 1.4	CrM
Stout et al. (62)	USA	RCT	Rowing/university	15	19.0 ± 2.0	CrM
Tarnopolsky et al. (67)	Canada	Randomized double-blind counterbalanced crossover	Recreational	12	21.9 ± 1.7	CrM
Cox et al. (31)	Australia	Double-blind matched-group controlled trial; allocation method not reported	Soccer/international	12	22.1 ± 5.4	CrM
Biwer et al. (72)	USA	Double-blind repeated-measures crossover	Soccer/university	8	NR	CrM
Kambis et al. (44)	USA	Non-randomized experimental	Recreational	22	20.3 ± 0.2	CrM
Lehmkuhl et al. (68)	USA	Double-blind RCT	Track and field/collegiate	12 eligible women within mixed cohort	Treatment groups 19.2–20.1	CrM; CrM + glutamine arm
Eckerson et al. (45)	USA	Randomized double-blind crossover	Recreational	10	22.0 ± 5.0 (19–34)	Not specified
Eckerson et al. (73)	USA	RCT	Recreational	30	21.0 ± 3.0	CrCi
Ferguson et al. (37)	Canada	RCT	Resistance-trained women	26	24.6 ± 3.4 (18–35)	CrM
Silva et al. (39)	Portugal	RCT	Swimming/national	16	CS: 16.3 ± 1.8; PL: 15.7 ± 1.2	CrM
Lim JY (63)	USA	Double-blind RCT	Volleyball/collegiate	36	20.6 ± 1.73 (19–26)	Not specified
Fukuda et al. (46)	USA	RCT	Recreational	26	21.0 ± 1.0 (18–24)	CrCi
Candow et al. (74)	Canada	Double-blind placebo-controlled trial	Physically active non-resistance-trained university students	24 eligible women within mixed cohort	21–28	CrM
Kresta et al. (49)	USA	RCT	Recreational	32	21.5 ± 2.8	CrM
Smith-Ryan et al. (47)	USA	RCT	Recreational	12	22.3 ± 2.5	DiCi
Morris et al. (69)	USA	Double-blind randomized trial	Habitually active adults	35 eligible women within mixed cohort	22 ± 2	Not specified
Ramírez-Campillo et al. (32)	Chile	RCT	Soccer/amateur	30	22.9 ± 2.5	CrM
Forbes et al. (48)	Canada	Randomized placebo-controlled trial	Recreationally active young females/HIIT	17	23 ± 4	CrM
Atakan et al. (40)	Turkey	RCT	Futsal/first-division, highly trained	30	19.83 ± 1.13	CrM
Azevedo et al. (50)	Brazil	Randomized double-blind placebo-controlled trial	Resistance-trained young women	28	25.5 ± 6.1	CrM
Brooks et al. (34)	USA	RCT	Dance/university	13	20.0 ± 1.0	CrM
Gordon et al. (33)	USA	Randomized double-blind crossover	Recreational	39	24.6 ± 5.9	CrM
Zahabi et al. (41)	Serbia	Quasi-experimental; purposive sampling; systematic random grouping	Freestyle wrestling/junior females	18	18.66 ± 0.93	CrM
Pereira et al. (42)	USA	Randomized controlled trial; no placebo	Beach volleyball/collegiate and professional	32	CrM 25.7 ± 5.6; control 29.6 ± 9.7	CrM gummies

Intervention protocols generally compared oral creatine supplementation with placebo or non-creatine control conditions, with creatine monohydrate the most commonly reported form when the supplement type was specified. Intervention duration and co-interventions varied across studies, including short loading protocols, multi-week supplementation, resistance-training interventions, sport training and laboratory exercise protocols. Study characteristics, including country, design, sport or training level, sample size, age and creatine form, are summarized in Table 1; supplementation protocols, outcome assessments and each study's synthesis role are summarized in Table 2.

**Table 2:** Supplementation protocols, outcomes, principal findings and synthesis status of included studies.

Study	Creatine regimen and study context	Outcomes assessed	Main findings	Synthesis status
Thompson et al. (70)	2 g/day; 6 weeks; Usual swim training schedule	100- and 400-m freestyle time; plantar-flexion exercise duration; muscle phosphocreatine and metabolite ratios; muscle oxygenation and recovery	Creatine did not improve swimming or plantar-flexion performance, muscle creatine-related metabolite ratios, oxygen supply, or aerobic/anaerobic metabolism relative to placebo	Narrative synthesis; no model-compatible female arm-level contrast
Grindstaff et al. (64)	7 g, 3 times/day; 9 days; usual swim training schedule	Three repeated 100-m freestyle swims; repeated 20-s arm-ergometer sprints; body mass, body composition, and total body water	In the mixed-sex cohort, creatine improved selected repeated-swim measures and first arm-ergometer-sprint work/power; body-composition and total-body-water differences were not significant	Narrative synthesis; mixed-sex results without female-specific estimates
Vandenberghe et al. (35)	5 g, 4 times/day for 4 days; then 2.5 g, twice/day for 10 weeks; 10 weeks plus loading; resistance training, 1 h three times/week	Maximal strength; intermittent arm-flexor exercise capacity; muscle phosphocreatine; body mass, body fat, and fat-free mass	Creatine augmented training-related gains in maximal strength, intermittent exercise capacity, and fat-free mass; body mass and body-fat changes were not clearly different from placebo	Primary multilevel meta-analysis; 3 effect sizes
Kirksey et al. (65)	0.3 g/kg/day; 6 weeks; preseason periodized track-and-field training	Countermovement jump height and power; repeated-cycle peak/average power, initial rate of power production, and total work; lean body mass	Across the mixed-sex cohort, creatine produced greater gains in countermovement-jump performance, cycle power/work, and lean body mass than placebo	Narrative synthesis; mixed-sex results without female-specific estimates
Ledford et al. (71)	5 g, 4 times/day; 5 days/condition; washout 95 ± 3 days	Peak power and work capacity during three Wingate tests; body mass	Creatine did not improve Wingate peak power or work capacity and did not produce a clear body-mass effect	Narrative synthesis; crossover data not pooled
Leenders et al. (57)	5 g, 4 times/day for 6 days; then 5 g, twice/day for 8 days; 2 weeks; Usual swim training schedule	Repeated 6 × 50-m and 10 × 25-yard swimming performance	Creatine did not improve repeated-swim performance in women; the reported performance benefit was confined to men	Narrative synthesis; graph-digitized outcome retained for sensitivity analysis
Brenner et al. (36)	5 g, 4 times/day for 7 days; then 2 g/day for 4 weeks; 5 weeks; Resistance training, 3 days/week	Bench-press strength; isokinetic knee-extension work and fatigue; body mass, body fat, and fat-free mass; blood lactate	Creatine enhanced bench-press strength gain and reduced skinfold-estimated body fat, but did not clearly improve knee-extension work/fatigue, fat-free mass, or lactate responses	Primary multilevel meta-analysis; 7 effect sizes
Haff et al. (66)	0.3 g/kg/day; 6 weeks; Preseason periodized track-and-field training	Countermovement-jump displacement; static-jump displacement and power/force-time measures; body mass and lean body mass	Across the mixed-sex cohort, creatine improved countermovement-jump performance and lean body mass, whereas most force-time measures did not show clear between-group effects	Narrative synthesis; mixed-sex results without female-specific estimates
Hamilton et al. (43)	5 g, 5 times/day; 7 days	Elbow-flexor and shoulder-internal-rotator peak strength, work capacity/fatigue, and velocity; body mass	Creatine improved the change in elbow-flexor work capacity/fatigue, but not peak elbow-flexor or shoulder-rotator strength, shoulder-rotator fatigue/velocity, or body mass	Primary multilevel meta-analysis; 8 effect sizes
Larson-Meyer et al. (38)	7.5 g, twice/day for 5 days; then 5 g/day, 5 days/week thereafter; 13 weeks including a 2-week no-supplement/no-training break; soccer and resistance training	Bench-press and full-squat 1RM; vertical jump; body mass, fat mass, and fat- and bone-free lean mass	Creatine produced greater bench-press and squat strength gains, but no clear additional effect on body mass or body-composition measures	Primary multilevel meta-analysis; 6 effect sizes
Stout et al. (62)	5 g, 4 times/day; 5 days	Physical working capacity at the fatigue threshold during incremental cycling; body mass	Creatine increased physical working capacity at the fatigue threshold relative to placebo (adjusted post-supplementation values, 186 vs. 155 W)	Primary multilevel meta-analysis; 2 effect sizes
Tarnopolsky et al. (67)	5 g, 4 times/day; 4 days/condition; washout 52 ± 6 days; usual activity	Repeated 30-s cycle power and lactate; dorsiflexor maximal voluntary contraction, fatigue, and stimulated torque; knee-extension torque; handgrip strength	Creatine increased peak/relative cycle power, dorsiflexor maximal voluntary contraction, and post-exercise lactate in the pooled cohort; the authors reported no sex-specific response	Narrative synthesis; mixed-sex crossover results without female-specific estimates
Cox et al. (31)	5 g, 4 times/day; 6 days; usual soccer training schedule	Repeated 20-m sprints, agility runs, and kicking accuracy; body mass; heart rate, lactate, and perceived exertion	Creatine increased body mass and improved selected repeated-sprint and agility trials, but not overall mean sprint/agility performance or kicking accuracy	Primary multilevel meta-analysis; 8 effect sizes
Biwer et al. (72)	0.075 g/kg, 4 times/day; 6 days/condition; washout at least 4 weeks	Treadmill time to exhaustion with high-intensity intervals; perceived exertion; blood lactate; body mass	Creatine did not improve time to exhaustion, perceived exertion, or lactate responses; body mass was unchanged in women, with only a nonsignificant tendency toward lower lactate	Narrative synthesis; crossover data not pooled
Kambis et al. (44)	0.125 g/kg fat-free mass, 4 times/day; 5 days; usual activity; no resistance training	Quadriceps isokinetic average power and time to peak torque; body mass, body fat, and fat-free mass	Creatine increased quadriceps extension/flexion average power and reduced extension time to peak torque; body-composition measures were unchanged	Primary multilevel meta-analysis; 3 effect sizes
Lehmkuhl et al. (68)	0.3 g/kg/day for 1 week; then 0.03 g/kg/day for 7 weeks; separate CrM + 4 g/day glutamine arm; 8 weeks; sport-specific track-and-field training	Body mass and lean body mass; vertical jump; repeated-cycle power, work, and initial rate of power production	Across the mixed-sex cohort, creatine alone and creatine plus glutamine increased body mass, lean body mass, and initial rate of power production relative to placebo; jump and average-power gains were not clearly different	Narrative synthesis; mixed-sex results without female-specific estimates
Eckerson et al. (45)	5 g, 4 times/day; 5 days/condition; washout 5 weeks; usual activity	Anaerobic working capacity	5 days of creatine increased anaerobic working capacity by 22.1% relative to placebo	Narrative synthesis; crossover data not pooled
Eckerson et al. (73)	5 g, 4 times/day; 6 days; usual activity pattern	Anaerobic working capacity and body mass after creatine, creatine phosphate, or placebo	Relative anaerobic working capacity was higher with creatine than placebo in the pooled analysis; among women, the 13% increase was not statistically significant, and body mass increased during loading	Primary multilevel meta-analysis; 3 effect sizes
Ferguson et al. (37)	0.075 g/kg, 4 times/day for 7 days; then 0.03 g/kg/day for 58 days; 9.5 weeks; resistance training, 4 days/week	Bench-press and incline-leg-press 1RM; repetitions across five sets at 70% 1RM; relative strength and training volume; total and regional body composition	Both groups gained strength and lean body mass during resistance training, but creatine did not provide an additional benefit over placebo	Primary multilevel meta-analysis; 8 effect sizes
Silva et al. (39)	5 g, 4 times/day; 3 weeks; usual swim training schedule	25-m swimming performance; hydrodynamic drag and coefficient; mechanical power; body mass, body fat, and lean mass	Between-group differences in swim performance and body composition were not clear; within the creatine group, hydrodynamic drag, drag	Primary multilevel meta-analysis; 8 effect sizes
			coefficient, and mechanical power requirement decreased	
Lim JY (63)	5 g, 4 times/day for 5 days; then 5 g/day; 10 weeks; conditioning program with weight training	Bench-press strength; vertical jump; body mass, lean body mass, and body fat	Creatine plus conditioning produced greater improvements in bench press, vertical jump, body mass, and lean body mass than conditioning alone; body fat did not clearly differ	Primary multilevel meta-analysis; 5 effect sizes
Fukuda et al. (46)	5 g, 4 times/day; 5 days	Anaerobic running capacity; body mass	Creatine did not clearly improve anaerobic running capacity in women and produced only a nonsignificant body-mass increase; the reported performance response occurred in men	Primary multilevel meta-analysis; 2 effect sizes
Candow et al. (74)	0.15 g/kg on 2 RT days/week or 0.10 g/kg on 3 RT days/week; 6 weeks; resistance training, 2 or 3 days/week	Elbow-flexor muscle thickness; chest-press and leg-press strength; body mass	Across the mixed-sex cohort, creatine taken on two or three training days per week produced similar gains and modestly increased elbow-flexor thickness; women gained less leg-press strength than men receiving creatine	Narrative synthesis; female-specific creatine-vs.-placebo estimates incomplete
Kresta et al. (49)	0.3 g/kg for 7 days; then 0.1 g/kg for 3 weeks; 4 weeks	Body composition and total body water; muscle phosphagens; repeated Wingate power and fatigue; lactate threshold, peak oxygen uptake, and time to exhaustion	Creatine did not show broad between-group effects on body composition, aerobic outcomes, or muscle phosphagens; an isolated benefit was observed for relative peak power in the second Wingate after 4 weeks	Primary multilevel meta-analysis; 9 effect sizes
Smith-Ryan et al. (47)	5 g, 4 times/day; 5 days	Maximal voluntary contraction; voluntary activation; evoked twitch properties; central and peripheral fatigue; perceived exertion; body mass	Five days of creatine did not alter force, voluntary activation, twitch properties, or central/peripheral fatigue relative to placebo	Primary multilevel meta-analysis; 15 effect sizes
Morris et al. (69)	3 g/day; 8 weeks; Habitual activity; exercise tests pre/post	Repeated 10-s Wingate sprint peak/mean power and decline slopes; 5-km time-trial performance	Across the mixed-sex cohort, creatine alone preserved peak- and mean-power slopes during repeated sprints and improved 5-km time-trial completion time; adding sodium bicarbonate conferred no additional benefit	Narrative synthesis; mixed-sex results without female-specific estimates
Ramírez-Campillo et al. (32)	5 g, 4 times/day for 7 days; then 5 g/day for 5 weeks; 6 weeks; usual soccer training plus plyometric training	Jump height/power and reactive-strength index; sprint and repeated-sprint performance; change-of-direction speed; endurance; body mass	Plyometric training improved several outcomes in both supplemented groups, with creatine producing greater gains in selected jump and repeated-sprint measures than placebo	Primary multilevel meta-analysis; 12 effect sizes
Forbes et al. (48)	0.3 g/kg/day for 5 days; then 0.1 g/kg/day for 23 days; 4 weeks; HIIT, 3 sessions/week	Peak oxygen uptake, ventilatory threshold, cycling time trial, body composition, and insulin sensitivity	High-intensity interval training improved cardiorespiratory fitness and time-trial performance in both groups, but creatine did not provide a clear additional benefit for performance or body composition	Primary multilevel meta-analysis; 13 effect sizes
Atakan et al. (40)	0.125 g/kg, twice/day; 7 days	10-, 20-, and 30-m sprint time; agility; leg strength; body mass	7 days of creatine improved sprint, agility, and leg-strength performance relative to placebo without a clear increase in body mass	Primary multilevel meta-analysis; 6 effect sizes
Azevedo et al. (50)	20 g/day in four 5-g doses for 7 days; 7 days supplementation; 4-week resistance-training period; resistance training, 3 sessions/week	Half-squat and leg-press repetition performance; ratings of perceived exertion; body mass; resting and exercise heart rate and blood pressure	Creatine increased half-squat and leg-press repetition performance and body mass. Ratings of perceived exertion were lower during the final training session, with no additional adverse or clinically relevant heart-rate or blood-pressure response	Primary multilevel meta-analysis; 6 effect sizes
Brooks et al. (34)	0.1 g/kg, once/day; 6 weeks; usual dance training	Body mass, lean mass, fat mass, and total body water; Wingate power; dance-specific performance; cognitive tests	Creatine increased total body water and lean mass, but did not produce clear treatment effects on exercise performance or cognitive outcomes	Primary multilevel meta-analysis; 29 effect sizes
Gordon et al. (33)	5 g, 4 times/day; 5 days per condition; at least 4-week washout; repeated-sprint cycling test	Repeated-sprint peak/average power and fatigue index; heart-rate variability across menstrual-cycle phases	Creatine improved fatigue index specifically in the high-hormone/luteal phase, but broad effects on peak/average power and heart-rate variability were not clear	Narrative synthesis; crossover data not pooled
Zahabi et al. (41)	10 g on training days; 6 weeks; strength training, 4 sessions/week	Body mass, body mass index, body fat, fat-free mass, and hypertrophy; one-repetition maximum, agility, muscular power, maximal oxygen uptake, and resting heart rate	In the source-reported analysis, creatine plus strength training improved strength, agility, power, selected body-size/hypertrophy measures, and maximal oxygen uptake; body fat, fat-free mass, and resting heart rate did not clearly differ	Primary multilevel meta-analysis; 9 effect sizes
Pereira et al. (42)	5 g/day CrM gummies; 10 weeks; maintained beach-volleyball training; no strict diet/exercise control	Countermovement jump, change-of-direction speed, and reaction time; body mass, lean mass, skeletal muscle mass, fat mass, body-fat percentage, and total body water	Creatine improved countermovement jump and change-of-direction speed and maintained fat mass/percentage while control values increased; reaction time and lean/total body mass did not clearly differ	Primary multilevel meta-analysis; 8 effect sizes

Across the systematic-review set, eligible outcomes covered exercise and sport performance, body composition, physiological outcomes and a small number of cognitive or reaction-time outcomes. Extracted source data contained performance-related outcomes from 24 studies, body-composition outcomes from 20 studies, physiological outcomes from 9 studies and cognitive or reaction-time outcomes from 2 studies.

### 3.3 Narrative synthesis of the systematic-review evidence

Table 2 presents the assessed outcomes and principal findings for all 34 included studies. Exercise and sport performance, body composition, physiological measures, cognition and reaction time were represented. Several studies reported outcomes in more than one domain.

#### 3.3.1 Exercise and sport performance

Exercise and sport performance results varied across tests and study designs. Favorable findings were reported for selected strength, jumping, sprint or repeated-sprint tests, fatigue resistance and sport-specific performance in female cohorts (31, 32, 35, 36, 38, 40–45, 49, 50, 62, 63). Several mixed-sex cohorts also reported favorable findings; female-specific estimates were unavailable (64–69). Other studies found no clear creatine effect on swimming or running performance, Wingate performance, neuromuscular fatigue, or adaptation to high-intensity interval training (34, 39, 46–48, 57, 70–73). Ferguson (37) found no clear additional benefit for resistance-training strength or training volume. Gordon (33)reported improved fatigue index during the high-hormone/luteal phase; peak and average power did not clearly change.

#### 3.3.2 Body composition

Body-composition findings also differed among studies. Increases in fat-free or lean mass, body mass, total body water, muscle thickness, maintenance of fat mass, or selected body-size measures were reported in some female and mixed-sex cohorts (31, 34–36, 41, 42, 50, 63, 65, 66, 68, 73, 74). Other studies found no clear between-group differences in body mass or measured body-composition outcomes (37–40, 43, 44, 46, 48, 49, 64, 71, 72).

#### 3.3.3 Physiological and cognitive outcomes

Physiological outcomes were less common and measured different processes. Muscle metabolites and oxygenation, lactate, neuromuscular activation and fatigue, cardiovascular measures, and insulin sensitivity showed no clear between-group effects in several studies (47–49, 70, 72). Azevedo (50) found no additional cardiovascular effect but reported lower perceived exertion during the final training session (50). Zahabi (41) reported increased maximal oxygen uptake and no clear change in resting heart rate. Pooled mixed-sex results from Tarnopolsky (67) showed increases in cycling and dorsiflexor outcomes; female-specific estimates were unavailable. Gordon (33) found a phase-specific improvement in fatigue index and no clear change in heart-rate variability. Cognitive performance in Brooks (34) and reaction time in Pereira (42) did not clearly improve.

### 3.4 Risk of bias

**Figure 2:**
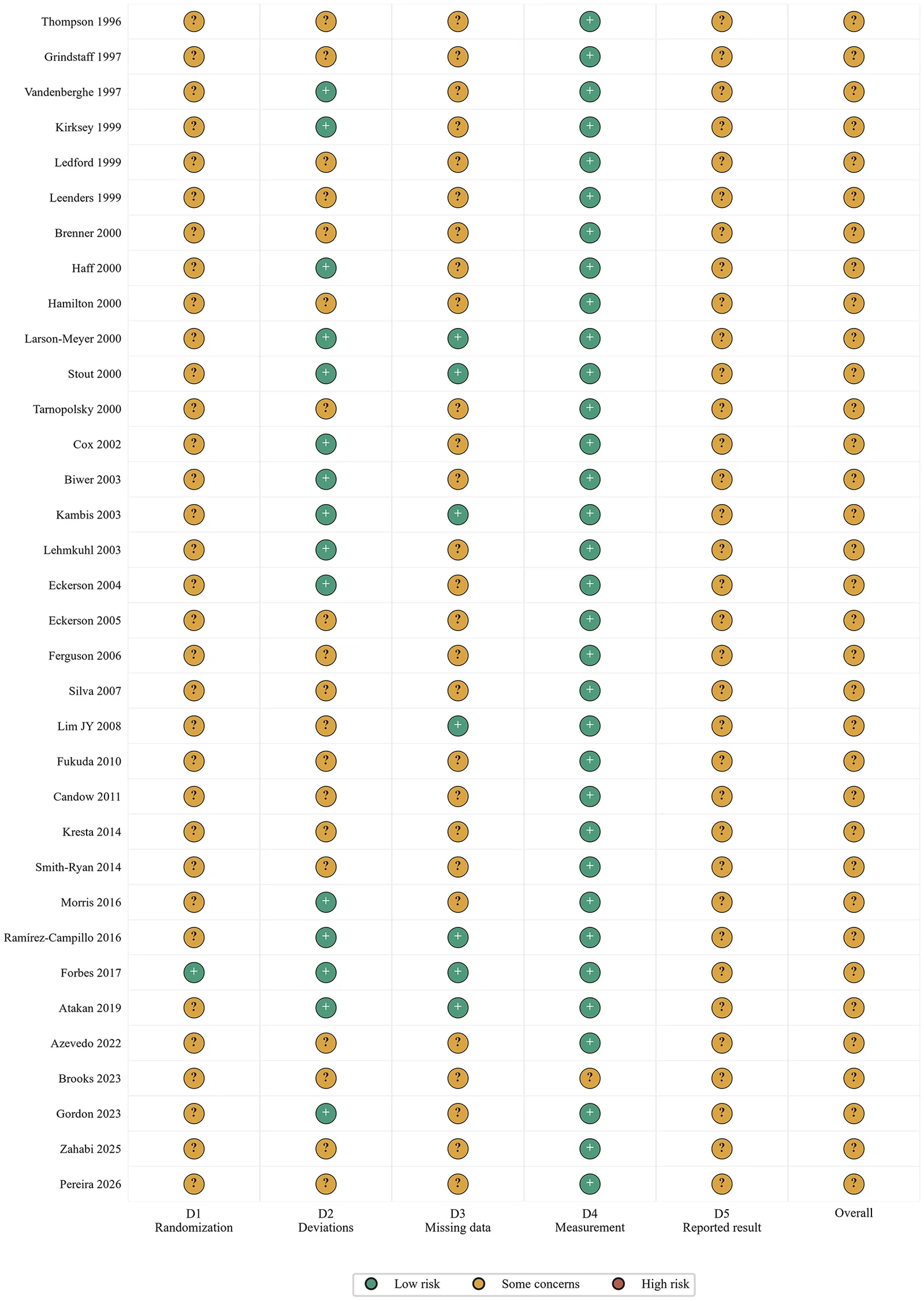
Risk of bias and design limitations across studies included in the systematic review. Randomized or allocated controlled trials were assessed using the Cochrane RoB 2 framework. Kambis 2003, the explicitly non-randomized experimental study, is shown for completeness using a design-informed appraisal and was not included in the 33-study RoB 2 domain-count summary. No-placebo and incomplete blinding or allocation reporting were considered when interpreting certainty.

Risk of bias and design limitations were assessed for all 34 studies included in the systematic review (Figure 2). No study clearly met low-risk criteria across all applicable appraisal domains. Most concerns reflected incomplete reporting of allocation, blinding, outcome completeness or prespecified reporting safeguards.

The RoB 2 domain summary was restricted to the 33 randomized or allocated controlled studies. In these studies, the randomization-process domain was judged as some concerns in 32 studies and low risk in 1 study. Deviations from intended interventions were judged as low risk in 14 studies and some concerns in 19 studies. Missing outcome data were judged as low risk in 6 studies and some concerns in 27 studies. Outcome measurement was judged as low risk in 32 studies and some concerns in 1 study. Selection of the reported result was judged as some concerns in all 33 studies. Kambis (44) was treated separately as an explicitly non-randomized experimental study and was interpreted with attention to confounding and participant-selection concerns.

The risk-of-bias pattern reflected reporting limitations. However, the presence of an explicitly non-randomized experimental study, no-placebo controlled design features in one recent study, and older trials with unclear allocation concealment or blinding were treated as reasons for caution in the certainty assessment. In many older trials, allocation sequence generation, allocation concealment, blinding details, analysis-plan prespecification or selective-reporting safeguards were incompletely described. Outcome measurement was generally less concerning because most included outcomes were laboratory, performance or body-composition measures with relatively structured assessment procedures. The selection-of-reported-result domain was the most persistent limitation because study protocols, trial registrations or predefined outcome hierarchies were usually unavailable.

### 3.5 Studies contributing to the primary meta-analysis

Twenty-one studies provided female-specific creatine-vs.-control contrasts suitable for the primary multilevel model (Table 2). Eight studies lacked female-specific or model-compatible arm-level data: Thompson (70), Grindstaff (64), Kirksey (65), Haff (66), Tarnopolsky (67), Lehmkuhl (68), Candow (74), and Morris (69). Four crossover studies were not pooled because the required within-participant correlations were unavailable: Ledford (71), Biwer (72), Eckerson (45), and Gordon (33). Leenders (57) was retained for narrative synthesis and graph-digitized sensitivity analysis.

Hedges g could be calculated for 171 extracted outcomes. The graph-digitized Leenders outcome was reserved for sensitivity analysis, leaving 170 effect sizes from 424 female participants in the primary multilevel dataset. This count differs from the Table 1 female/eligible n because the primary model counted only participants contributing to analyzable creatine- vs.-control contrasts, excluding additional intervention arms, unused comparison groups and participants without model-compatible contrasts. The primary model included 68 exercise and sport performance effects, 62 body-composition effects, 33 physiological effects and 7 cognitive or reaction-time effects. Cognitive or reaction-time effects were retained in the overall model as secondary eligible outcomes but were not fitted as a separate domain model because they were reported by only two studies.

The number of effects per study varied substantially. Some studies contributed only two or three eligible contrasts. Others contributed larger outcome sets across multiple tests, time points, or body-composition variables. Brooks (34) contributed the largest number of extractable effects, largely because it reported multiple body-composition and related outcomes. Smith-Ryan (47), Forbes (48), and Ramírez-Campillo (32) also contributed multiple effects across performance or training-adaptation outcomes. The primary synthesis therefore retained effect-level data in a multilevel model. Selecting one endpoint per study would have discarded eligible information.

### 3.6 Primary multilevel meta-analysis

**Figure 3:**
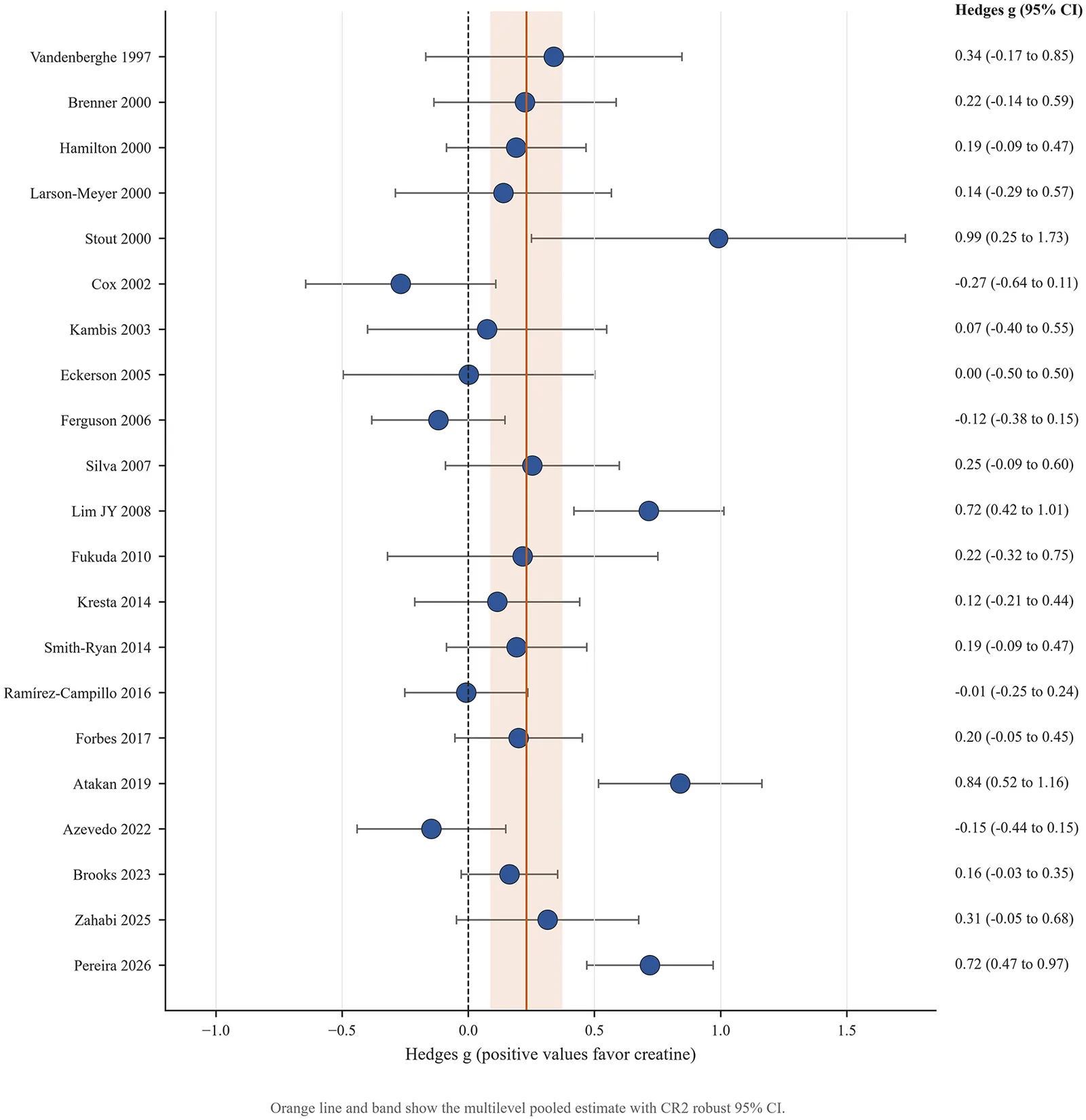
Overall multilevel forest plot. Study-level estimates are shown for the primary multilevel meta-analysis of creatine supplementation across eligible outcomes. The pooled estimate is presented with a CR2 robust 95% confidence interval.

The primary multilevel model included 21 studies and 170 effect sizes (Figure 3 and Table 3). Creatine supplementation was associated with a positive overall effect across eligible outcomes (Hedges g = 0.23, CR2 robust 95% CI 0.09 to 0.37, *p* = 0.003). The conventional model-based interval was similar (95% CI 0.10 to 0.36).

**Table 3:** Primary and domain-specific multilevel meta-analysis results.

Analysis	Studies	Effect sizes	Hedges g	CR2 robust 95% CI	Robust SE	Robust df	*p* value	Model note
Overall eligible outcomes	21	170	0.23	0.09 to 0.37	0.068	19.0	0.003	Primary multilevel model; σ^2^ study = 0.061, σ^2^ effect = 0.024, random-effects ICC = 0.72
Exercise and sport performance	18	68	0.30	0.06 to 0.54	0.114	15.5	0.019	Domain-specific multilevel model with CR2 robust confidence interval
Body composition	18	62	0.24	0.06 to 0.43	0.084	14.6	0.011	Domain-specific multilevel model with CR2 robust confidence interval
Physiological outcomes	8	33	0.10	−0.17 to 0.38	0.102	4.5	0.361	Domain-specific multilevel model with CR2 robust confidence interval

Study-level visual aggregation showed heterogeneity (I^2^ = 70.95%; Q = 68.93, df = 20, *p* < 0.001). The full multilevel dataset also showed residual heterogeneity (Q = 239.09, df = 169, *p* < 0.001). Estimated random-effect variance was 0.061 between studies and 0.024 among effects within studies. The study-level component represented 72.0% of total estimated random-effect variance (random-effects ICC or variance-partition coefficient = 0.72).

### 3.7 Domain-specific syntheses

**Figure 4:**
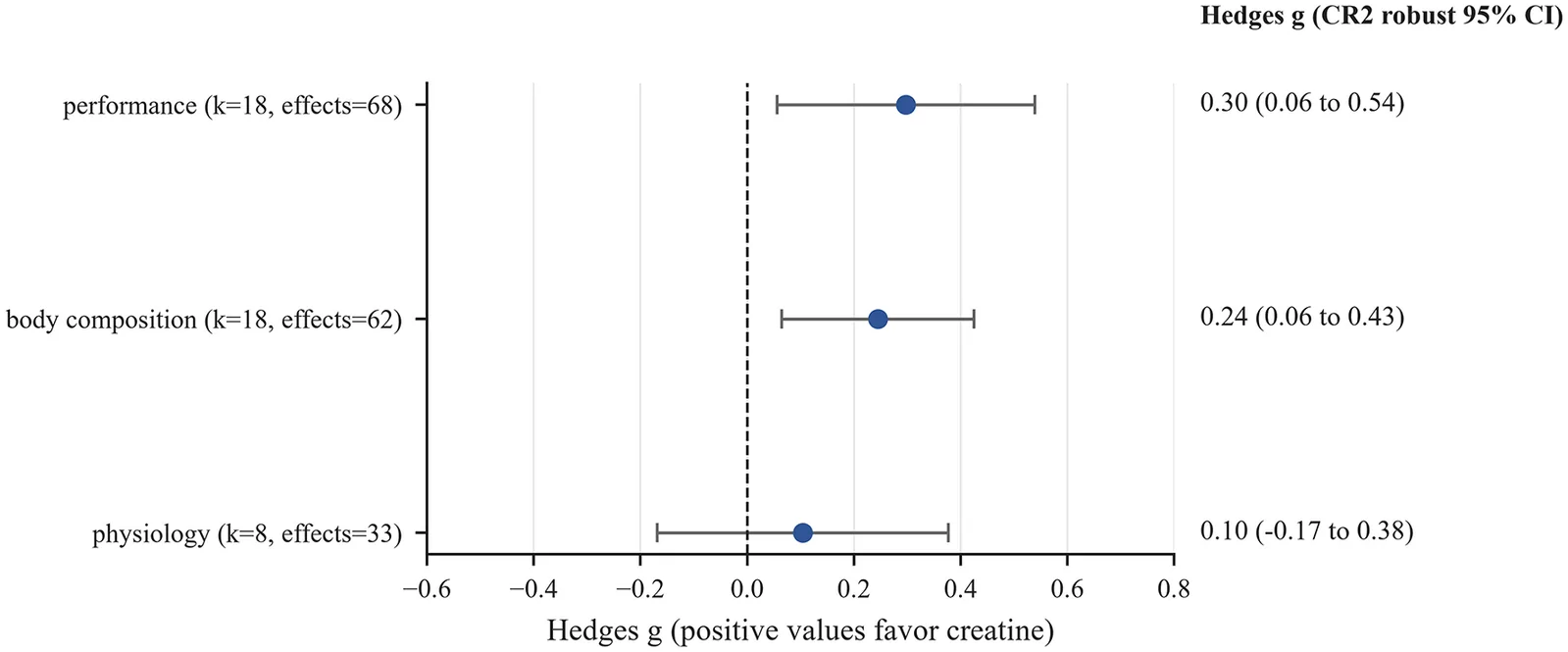
Domain-specific pooled effects. Multilevel pooled estimates are shown for exercise and sport performance, body composition, and physiological outcomes. Estimates are presented as Hedges g with CR2 robust 95% confidence intervals.

Domain-specific estimates varied by outcome class (Figure 4 and Table 3). Exercise and sport performance outcomes showed a positive pooled effect (18 studies, 68 effect sizes; Hedges g = 0.30, 95% CI 0.06 to 0.54, *p* = 0.019). Body-composition outcomes also showed a positive pooled effect (18 studies, 62 effect sizes; Hedges g = 0.24, 95% CI 0.06 to 0.43, *p* = 0.011). Physiological outcomes did not show a clear pooled effect (8 studies, 33 effect sizes; Hedges g = 0.10, 95% CI −0.17 to 0.38, *p* = 0.361).

Residual heterogeneity differed by domain. It was detected for performance outcomes (Q = 129.78, df = 67, *p* < 0.001), while the tests for body composition (Q = 75.00, df = 61, *p* = 0.107) and physiological outcomes (Q = 28.27, df = 32, *p* = 0.656) were not statistically significant. In the *post hoc* duration strata, studies lasting ≤ 7 days yielded g = 0.20 (95% CI −0.13 to 0.53; 9 studies, 53 effects), and studies lasting >7 days yielded g = 0.25 (95% CI 0.08 to 0.43; 12 studies, 117 effects). These estimates were descriptive, and no between-stratum test was performed.

### 3.8 Sensitivity analyses and certainty of evidence

**Figure 5:**
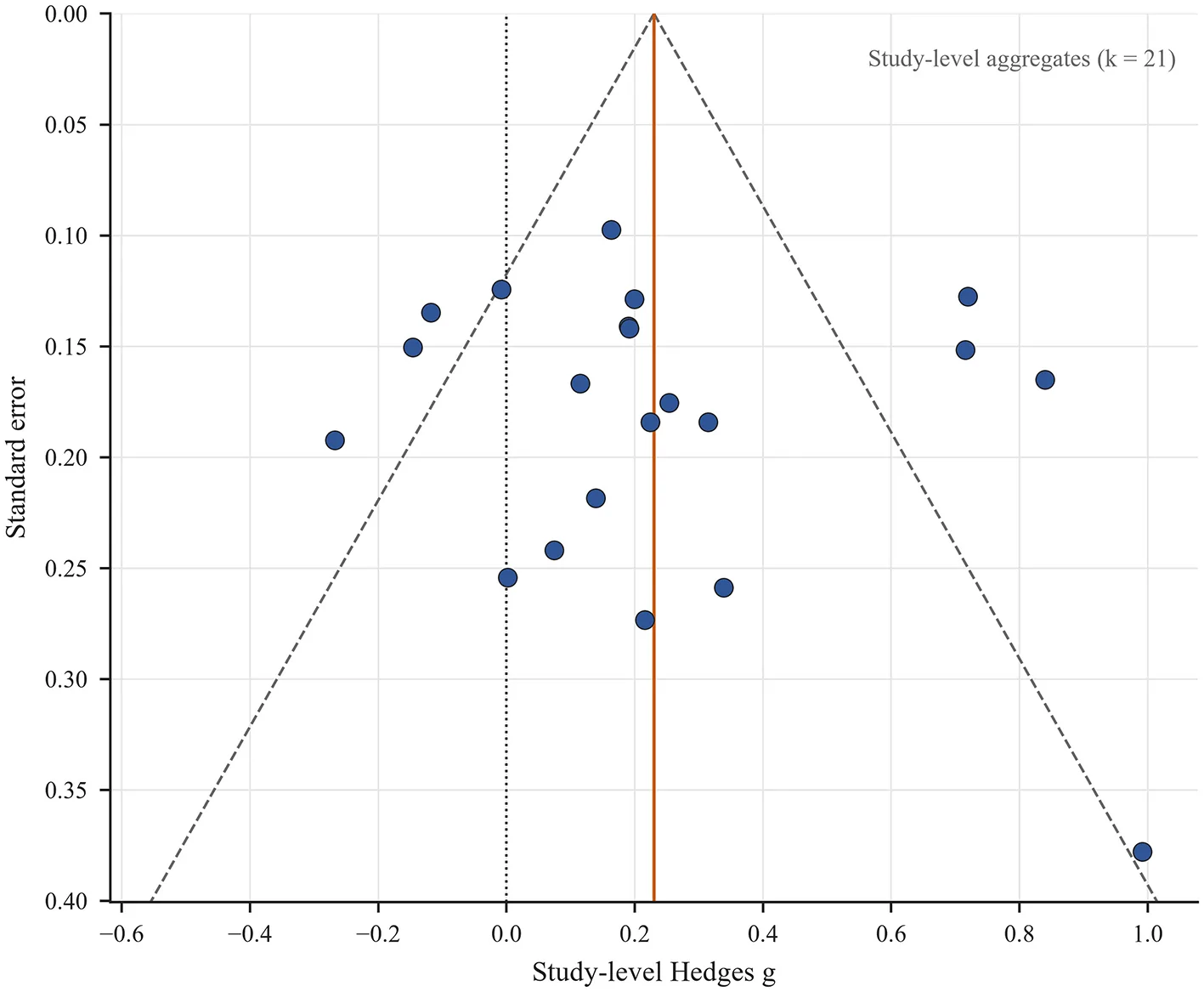
Funnel plot of study-level aggregated effects. Each point is the inverse-variance weighted aggregate of eligible primary effects within one study. Dashed lines show pseudo 95% limits around the conventional study-level random-effects estimate; the orange line marks that estimate. The plot and study-level regression (z = 0.67, *p* = 0.505) are exploratory because each aggregate combines dependent outcomes and only 21 studies were available.

The primary estimate was stable across sensitivity analyses (Table 4). Including the duplicate-digitized Leenders outcome (57) gave g = 0.23 (22 studies, 171 effects; 95% CI 0.09 to 0.37, p = 0.003). Excluding reconstructed percentage-change rows gave g = 0.25 (20 studies, 158 effects; 95% CI 0.10 to 0.39, p = 0.003). Removing effects with absolute Hedges g greater than 3 did not change the rounded estimate. Leave-one-study-out estimates ranged from 0.20 to 0.25; all lower CR2 confidence limits remained above zero. Assumed within-study sampling correlations of 0.30, 0.50 and 0.80 produced estimates of 0.23, 0.23 and 0.22, with lower confidence limits of 0.08, 0.07 and 0.07. The conventional random-effects model of 21 study-level aggregates gave g = 0.23 (95% CI 0.10 to 0.36). The study-level funnel plot is shown in Figure 5; asymmetry regression gave z = 0.67 and *p* = 0.505. This diagnostic remains exploratory because study aggregates contain correlated outcomes and the test is underpowered at k = 21.

**Table 4:** Sensitivity analyses and heterogeneity diagnostics.

Analysis	Studies	Effect sizes or units	Hedges g	95% CI or diagnostic	*p* value
Primary analysis	21	170	0.23	0.09 to 0.37	0.003
Including duplicate-digitized Leenders outcome (57)	22	171	0.23	0.09 to 0.37	0.003
Excluding reconstructed percentage-change rows	20	158	0.25	0.10 to 0.39	0.003
Excluding effects with absolute Hedges g >3	21	170	0.23	0.09 to 0.37	0.003
Assumed within-study sampling r = 0.30	21	170	0.23	0.08 to 0.39	0.005
Assumed within-study sampling r = 0.50	21	170	0.23	0.07 to 0.39	0.006
Assumed within-study sampling r = 0.80	21	170	0.22	0.07 to 0.38	0.007
Study-level aggregate random-effects model	21	21	0.23	0.10 to 0.36	<0.001
Leave-one-study-out range	20 per model	141 to 168 per model	0.20 to 0.25	lower limits 0.06 to 0.11	0.001 to 0.007
Duration ≤ 7 days; descriptive	9	53	0.20	−0.13 to 0.53	0.190
Duration >7 days; descriptive	12	117	0.25	0.08 to 0.43	0.009
Study-level heterogeneity	21	21	–	Q (20) = 68.93; I^2^ = 70.95%	<0.001
Overall multilevel heterogeneity	21	170	–	Q (169) = 239.09	<0.001
Performance residual heterogeneity	18	68	–	Q (67) = 129.78	<0.001
Body-composition residual heterogeneity	18	62	–	Q (61) = 75.00	0.107
Physiological residual heterogeneity	8	33	–	Q (32) = 28.27	0.656

CR2 robust confidence intervals are shown for multilevel effect estimates. The study-level aggregate row uses a conventional random-effects interval. Sampling-correlation models imposed compound-symmetric covariance blocks within studies. Duration strata were *post hoc* and descriptive; no between-stratum test was performed. The Leenders row uses the reconciled result of two independent coordinate-calibration passes. Heterogeneity rows report Q tests and are not pooled effects.

Certainty of evidence was very low for the overall and physiological estimates and low for exercise and sport performance and body composition (Table 5). Overall indirectness was serious because the global model combined different outcome constructs, exercise and training contexts, protocols and baseline training states. The performance rating reflected residual inconsistency across tests and protocols; the physiological rating also reflected imprecision. GRADE describes confidence that an estimated effect is close to the corresponding true effect. Confidence was therefore low for the positive performance and body-composition estimates and very low for the global and physiological estimates.

**Table 5:** GRADE summary of findings.

Outcome	Evidence base	Pooled effect	Main downgrading concerns	Certainty
Overall effect across eligible outcomes	21 studies; 170 effects	Hedges g 0.23 (0.09 to 0.37); *p* = 0.003	RoB/design concerns; heterogeneity; cross-domain indirectness	Very low
Performance outcomes	18 studies; 68 effects	Hedges g 0.30 (0.06 to 0.54); *p* = 0.019	RoB/design concerns; heterogeneity across tests and protocols	Low
Body-composition outcomes	18 studies; 62 effects	Hedges g 0.24 (0.06 to 0.43); *p* = 0.011	RoB/design concerns; heterogeneous measures and protocols	Low
Physiology outcomes	8 studies; 33 effects	Hedges g 0.10 (−0.17 to 0.38); *p* = 0.361	RoB/design concerns; heterogeneity; indirectness; imprecision	Very low

## 4 Discussion

### 4.1 Principal findings

In this systematic review and multilevel meta-analysis, creatine supplementation was associated with a small positive average across eligible outcomes in females engaged in exercise or training. The global estimate serves as a directional summary across constructs; the domain estimates provide the main scientific interpretation. Exercise and sport performance and body composition showed positive estimates, while the physiological estimate remained uncertain. Results were stable across data-source, influence and sampling-correlation analyses. Low or very-low certainty limits confidence in the proximity of these estimates to the true effects. Interpretation should remain cautious and confined to the outcome classes and study contexts represented in the available evidence.

The review combined two evidence layers. The systematic review included 34 studies and 692 female or female-eligible participants, capturing the full experimental literature that met the eligibility criteria. The primary meta-analysis included 21 studies, 170 model-compatible female-specific effects and 424 analyzable participants. Mixed-sex reporting, crossover or repeated-measures designs, graph-only outcomes and incomplete variance data accounted for most of the difference. Those studies informed the narrative synthesis and remained outside the primary independent-arm model.

The pooled performance and body-composition estimates, g = 0.30 and g = 0.24, are small standardized effects. Their practical importance depends on the outcome scale, baseline performance and competitive setting. A small change can matter when elite performance margins are narrow, yet a standardized average across sprint time, strength, jump tests and body-composition measures cannot be converted into a common number of seconds, kilograms or competitive places. Statistical significance therefore provides limited information about sport-specific importance without the original measurement scale and a task-relevant threshold.

### 4.2 Performance outcomes

The performance estimate is consistent with the broader ergogenic rationale for creatine. Earlier meta-analysis linked supplementation to body-composition and performance outcomes in wider samples (2), and later syntheses reported favorable effects for lower-limb strength (4), resistance-training strength gains in adults younger than 50 years (6), and repeated-sprint ability (75). Benefits in broader performance studies are concentrated in intermittent, repeated and high-intensity tasks (76–81). Female-specific examples include sport-specific running in elite soccer players (31), maximal quadriceps contraction (44), plyometric and sprint outcomes in soccer players (32), sprint and agility in futsal players (40), resistance-training outcomes in junior wrestlers (41), and beach-volleyball performance (42).

This pattern fits the phosphocreatine mechanism (7–9): greater phosphagen availability is most relevant to rapid energy turnover, repeated high-intensity efforts, strength expression and training quality. Weaker or null results in other exercise modes are biologically plausible when phosphocreatine availability is not the main performance constraint (48, 82). The pooled performance estimate remains an average across distinct tasks, doses and training states.

Female-specific mechanistic interpretation remains provisional. Dietary creatine exposure and baseline muscle creatine may influence the capacity for tissue uptake (10–14, 22–25). Reproductive hormones also affect fluid regulation, substrate use and neuromuscular function, creating plausible variation across menstrual phases, contraceptive use and hormonal transitions. The included trials rarely measured baseline muscle creatine, diet, menstrual phase or contraceptive status, and most participants were young adults. The present data cannot identify these factors as modifiers of supplementation response. Their main implication is a reporting requirement for future trials.

### 4.3 Body composition

The positive body-composition estimate needs careful interpretation. Broader syntheses indicate that creatine can increase lean body mass or fat-free-mass-related outcomes, especially with resistance training (5, 83). In active female studies, body-composition endpoints combine biological tissue change with measurement-sensitive outcomes. DXA-derived lean mass and total body water can shift after supplementation in female collegiate dancers (34). Short-term increases in fat-free mass may partly reflect water-related changes, with limited evidence for concurrent contractile-tissue accretion (84, 85).

The body-composition estimate does not establish muscle hypertrophy across all female exercise or training populations. It indicates that creatine can shift body-composition measures, with the contributions of water, lean tissue and training adaptation depending on protocol duration, co-intervention and measurement method. Supplement timing and training context may modify these measurements when creatine accompanies resistance training (86, 87). Female athletes may evaluate these changes in relation to performance, weight categories, aesthetics and supplement acceptability. Future studies should distinguish short-term hydration changes from longer-term tissue adaptation and report the measurement method for each endpoint.

### 4.4 Physiological and secondary outcomes

The lack of a clear pooled physiological effect does not contradict the bioenergetic rationale for creatine. It reflects the narrower and more heterogeneous physiological evidence base in this review. Creatine supplementation can increase muscle creatine content in healthy young adults (12), and phosphorus magnetic resonance studies support links between creatine availability and phosphocreatine-related muscle metabolism (7, 8). The physiological outcomes extracted here covered a smaller and less coherent set of biomarkers and exercise-physiology endpoints. Some physiological responses may depend on training stimulus, dose timing, baseline creatine status or diet, none of which were consistently measured across studies. Previous mixed-context studies have also reported null or unclear physiological adaptations during aerobic or submaximal training settings (82, 88).

Secondary cognitive or reaction-time outcomes should be treated as exploratory. Creatine has been studied in relation to brain performance (89), cognitive function in young adults (90), cognition in older adults or vegetarians (91, 92), mental fatigue and visuomotor performance (93), corticomotor excitability and cognition under oxygen deprivation (94), brain health (95), and cognitive performance in a randomized trial (96). In the present dataset, only two studies contributed cognitive or reaction-time effects. This was not enough to support a separate domain model. The present findings should therefore not be used to make cognitive claims for females in exercise or training contexts. They indicate only that cognitive or reaction-time outcomes remain underdeveloped in the female creatine literature relevant to exercise and training.

### 4.5 Relation to safety and practical use

This review focused on efficacy outcomes. Safety context remains relevant because creatine use in sport is discussed alongside hydration, renal function and body-mass concerns. Earlier safety reviews and controlled sport studies have not supported common claims of renal harm, thermoregulatory impairment or excess clinical risk under studied conditions (97–100). Controlled data in type 2 diabetes (101) and recent reviews (102) also provide no clear renal-safety signal. Real-world observations in women's football provide additional context (28). Pediatric and adolescent discussions emphasize context-specific dosing, supervision and complete adverse-event reporting (103).

These safety references provide context and are not direct evidence from the present meta-analysis. Most eligible female trials were too small and too short to detect uncommon adverse events, and adverse-event reporting was inconsistent. Safety and tolerability should remain study-level priorities across training loads, menstrual status, dietary patterns and supplement-use practices. Future female trials should report efficacy, adherence, gastrointestinal symptoms, body-mass concerns, hydration-related events and reasons for discontinuation.

### 4.6 Certainty and methodological limitations

Several limitations define the boundary of these findings. First, risk-of-bias appraisal was limited by incomplete trial-method reporting and by the presence of one non-randomized experimental study within an evidence base otherwise dominated by controlled trials. These concerns were driven mainly by incomplete reporting of randomization, allocation concealment, analysis planning and selective-reporting safeguards. Outcome measurement was generally less problematic, but the absence of prespecified outcome hierarchies made selective reporting difficult to rule out. This explains why GRADE certainty could not be rated high even when some pooled confidence intervals excluded the null.

Second, variation was expected because the evidence base included different sports, training backgrounds, doses, loading and maintenance protocols, intervention durations, co-interventions and outcome definitions. Residual heterogeneity was concentrated in the performance domain; body-composition and physiological residual tests were not statistically significant. Duration strata produced overlapping estimates, so the available data did not isolate duration as an explanatory factor. Expected variation does not by itself invalidate a pooled estimate. The interpretive limitation is the remaining uncertainty about which performance tasks, protocols and participant groups account for the observed dispersion. Third, 13 systematic-review studies could not enter the primary model because of incomplete female-specific data, crossover structures, graph-only outcomes or other incompatibilities. Fourth, publication-bias assessment remained exploratory at 21 study-level aggregates.

The multilevel model addressed dependence through study and within-study random effects, with 72% of estimated random-effect variance located between studies. The primary model could not reconstruct unknown sampling covariances among outcomes. CR2 inference clustered effects by study, and additional models with assumed within-study correlations from 0.30 to 0.80 produced similar estimates. Retaining multiple outcomes used more eligible evidence and increased the importance of transparent classification, direction coding and extraction rules. Domain estimates, sensitivity analyses, risk-of-bias judgments and GRADE ratings should accompany interpretation of the global estimate.

Finally, registration occurred after completion of the primary synthesis and could not constrain review and analytic decisions prospectively. The publicly archived extraction files, decision rules, analysis code and sensitivity analyses improve transparency. These materials do not provide the safeguards of prospective protocol registration.

### 4.7 Implications for future research

Practical consideration of creatine for females engaged in exercise or training should remain conditional on the intended outcome, training stimulus and supplementation protocol. The current evidence does not justify a universal recommendation or firm expectations for a specific sport or outcome.

Future trials should move beyond small short-term designs and prespecify primary outcomes, report female-specific arm-level data, document menstrual or hormonal context where relevant, measure dietary pattern and baseline creatine exposure, and distinguish water-related body-composition changes from training-related tissue adaptation. Studies addressing recovery should separate performance recovery from indirect muscle-damage markers, because broader reviews suggest that creatine effects on exercise-induced muscle damage and recovery markers are not interchangeable with sport-performance benefits (104, 105). Studies that include both males and females should report sex-stratified means, standard deviations and sample sizes for each intervention arm. Crossover and repeated-measures studies should report within-participant correlation structures or sufficient paired data to enable synthesis. Until such reporting improves, multilevel meta-analysis can retain more evidence than single-endpoint synthesis, but it cannot correct for missing female-specific data or unclear trial reporting.

These reporting priorities are not merely technical. They determine whether future reviews can distinguish between true absence of effect, insufficient statistical power, poor outcome alignment and incomplete reporting. For females in exercise, sport or structured training contexts, the most useful next generation of trials would align supplementation protocol, sport demand and primary outcome before data collection, then report enough information for sex-specific synthesis. Such studies would make it possible to move from low-certainty average effects toward clearer sport-specific and outcome-specific recommendations.

## 5 Conclusion

Available female-specific evidence suggests possible small effects on selected strength, power, high-intensity performance and lean-mass-related measures. Substantial heterogeneity, methodological limitations and low or very-low certainty prevent firm sport-specific or outcome-specific conclusions. Physiological outcomes were uncertain, and cognitive or reaction-time data were too sparse for a separate conclusion. Practical decisions should remain conditional on the intended outcome, training stimulus and supplementation protocol because the evidence does not warrant a universal recommendation. Future studies should prioritize female-specific reporting, prespecified outcome hierarchies, transparent safety and adherence data, and body-composition methods that distinguish short-term water shifts from longer-term tissue adaptation.

## Data Availability

Publicly available datasets were analyzed in this study. This data can be found here: Repository name: Open Science Framework (OSF) Direct link: https://doi.org/10.17605/OSF.IO/G9ZTR Accession number/identifier: OSF.IO/G9ZTR.
